# Myeloid Immune Cells CARrying a New Weapon Against Cancer

**DOI:** 10.3389/fcell.2021.784421

**Published:** 2021-12-10

**Authors:** Rodrigo Nalio Ramos, Samuel Campanelli Freitas Couto, Theo Gremen M. Oliveira, Paulo Klinger, Tarcio Teodoro Braga, Eduardo Magalhães Rego, José Alexandre M. Barbuto, Vanderson Rocha

**Affiliations:** ^1^ Laboratory of Medical Investigation in Pathogenesis and Directed Therapy in Onco-Immuno-Hematology (LIM-31), Departament of Hematology and Cell Therapy, Hospital das Clínicas HCFMUSP, Faculdade de Medicina, University of São Paulo, São Paulo, Brazil; ^2^ Instituto D’Or de Ensino e Pesquisa, São Paulo, Brazil; ^3^ Fundação Pró-Sangue–Hemocentro de São Paulo, São Paulo, Brazil; ^4^ Department of Pathology, Federal University of Parana, Curitiba, Brazil; ^5^ Graduate Program in Biosciences and Biotechnology, Instituto Carlos Chagas, Fiocruz-Parana, Curitiba, Brazil; ^6^ Departamento de Imunologia, Instituto de CienciasBiomedicas, Universidade de Sao Paulo, São Paulo, Brazil; ^7^ Churchill Hospital, Department of Hematology, University of Oxford, Oxford, United Kingdom

**Keywords:** CAR (chimeric antigen receptor), monocyte, myeloid cells, solid tumor (malignancy and long term complications), macrophages, dendritic cell (DC)

## Abstract

Chimeric antigen receptor (CAR) engineering for T cells and natural killer cells (NK) are now under clinical evaluation for the treatment of hematologic cancers. Although encouraging clinical results have been reported for hematologic diseases, pre-clinical studies in solid tumors have failed to prove the same effectiveness. Thus, there is a growing interest of the scientific community to find other immune cell candidate to express CAR for the treatment of solid tumors and other diseases. Mononuclear phagocytes may be the most adapted group of cells with potential to overcome the dense barrier imposed by solid tumors. In addition, intrinsic features of these cells, such as migration, phagocytic capability, release of soluble factors and adaptive immunity activation, could be further explored along with gene therapy approaches. Here, we discuss the elements that constitute the tumor microenvironment, the features and advantages of these cell subtypes and the latest studies using CAR-myeloid immune cells in solid tumor models.

## Introduction

The myeloid immune cell compartment is composed by distinct cell subtypes that present a variety of functions once they have differentiated and maturated at the periphery. Within this compartment, besides the other cell types, the mononuclear phagocyte cells include subsets of monocytes, macrophages and dendritic cells (DC).

Monocytes are mostly found in the blood and macrophages are exclusively found in the tissues, while DC subsets can be found both in circulation and tissues.

Many phenotypic characteristics of DC associated with the induction of various T-cell response patterns have been described. It allows, for example, the association of the conventional Dendritic Cell 1 (cDC1) phenotype with the induction of CD8+ cytotoxic T lymphocytes (CTL) (Bachem et al., 2010), the conventional Dendritic Cell 2 (cDC2) phenotype with that of various Th subtypes (Segura et al., 2005; Rojas et al., 2017) but also of Treg cells (Watchmaker et al., 2014), while the plasmacytoid Dendritic Cell (pDC) phenotype has been mostly associated with the production of type I interferon (Reizis et al., 2011). However, this is an incomplete picture which is quickly being filled outand much still needs to be determined before one can predict the *in vivo* response from any DC phenotype introduced in the system.

Most recently, distinct groups have described other DC subset such as inflammatory-DCs (Segura et al., 2013) and type 3 DC (DC3) (Dutertre et al., 2019; Bourdely et al., 2020).

Several studies have highlighted the critical role of myeloid immune cells during tumor growth and metastasis, as reviewed by Engblom and collaborators (Engblom et al., 2016). Collective findings have put evidence on the association of tumor-associated macrophages (TAMs) with poor patients’ outcome for distinct tumor types (Medrek et al., 2012; Ino et al., 2013; Reinartz et al., 2014; Yang et al., 2018; Ramos et al., 2020; Guo et al., 2021). In contrast, mature DC subsets are currently associated to good prognosis, mostly due to their anti-tumoral role by stimulating T cell responses (Ladányi et al., 2007; Goc et al., 2014; Truxova et al., 2018). Also, an important role of type I IFNs has been discussed as being critical for the innate and adaptive immunity cross-talk (Demaria et al., 2019). Nucleic-acid-sensing cytosolic receptors, such as cGAS-STING pathways, found in the tumor microenvironment may trigger the production of type I IFNs that promote activation of NK cells, which subsequently stimulate DCs and T lymphocytes and anti-tumoral responses.

Numerous studies have reported the use of distinct strategies to overcome the immunosuppressive tumor microenvironment via the stimulation of myeloid immune cells (Chaib et al., 2020; Neophytou et al., 2020). These approaches include: the recruitment of a new wave of immune cells; the stimulation of cells via agonists/ligands; cytokine-based treatments; blockage of receptors by monoclonal antibodies and drug-mediated reprogramming of cells.

Very recently, some reports have described the insertion of chimeric antigen receptor (CAR) on macrophages (Klichinsky et al., 2020; Morrissey et al., 2018; W. Zhang et al., 2019; Zhang L. et al., 2020), an already known technology used for T cells (CAR-T) and natural killer cells (CAR-NK). CAR-T and CAR-NK cell therapies have achieved encouraging results in hematological tumors (Waldman et al., 2020). There has been a rapid growth of published data associated with CAR-T cells. Around 700 active clinical trials can be found at ClinicalTrials.gov database using CAR-T as a treatment modality. Most studies focus on hematologic malignancies, while 45 studies are related to solid tumor treatment. The inherent cytotoxicity of natural killer (NK) cells against tumors and their potential as an “off-the-shelf” cell therapy product have encouraged several clinical trials using CAR expressing NK cells to treat a number of malignant diseases (Xie et al., 2020). There are currently 19 active CAR-NK registered clinical trials, most of them targeting CD19+ hematological malignancies.

The myeloid lineages, especially the mononuclear phagocyte system, present an auspicious future, considering their functional capacity, which includes phagocytosis, antigen presentation, T cell co-stimulation, extracellular matrix remodeling and infiltration into the tumor microenvironment (Anderson et al., 2021; Chen et al., 2021). Despite technical issues concerning transfection, gene delivery and stable expression of CAR on myeloid immune cells (Dokka et al., 2000; Stroh et al., 2010; Keller et al., 2018), preliminary *in vitro* and pre-clinical results using CAR-Macrophages (CAR-Mac) have shown exciting data. We discuss here some important features of monocytes, DCs and macrophages for cancer therapy and the newly reported studies engineering myeloid cells to express CAR.

## Ontogeny of Myeloid Immune Cells

The mononuclear phagocyte system was originally described as bone marrow-derived myeloid cells that circulate in the blood as monocytes and reside in tissues as macrophages in both the steady state and inflammation (Furth and Cohn 1968). It is now known that different progenitors can give rise to several cell subsets with distinct phenotypes and particular biological functions. Additionally, migration to tissues and the differentiation of lineage-committed progenitors might be influenced by the surrounding microenvironment such as the inflammatory milieu (Serbina et al., 2008). Monocytes respond to their environment by differentiating into a variety of macrophages and DC-like cells to mount specific functional programs (Taylor and Gordon 2003). The generation and development of monocytes, macrophages and DCs is driven by the association of specific cytokines and growth factors with receptors expressed in hematopoietic stem cell-derived precursors (Sasmono et al., 2003). The bone marrow-derived progenitors are responsible for the renewal of a substantial set of myeloid cells, although many tissue-resident macrophages and DCs subsets (microglia and Langerhans cells) seem to be able to self-renewal, and, thus, independent of this developmental pathway (Ajami et al., 2007; Merad and Manz 2009).

**FIGURE 1:**
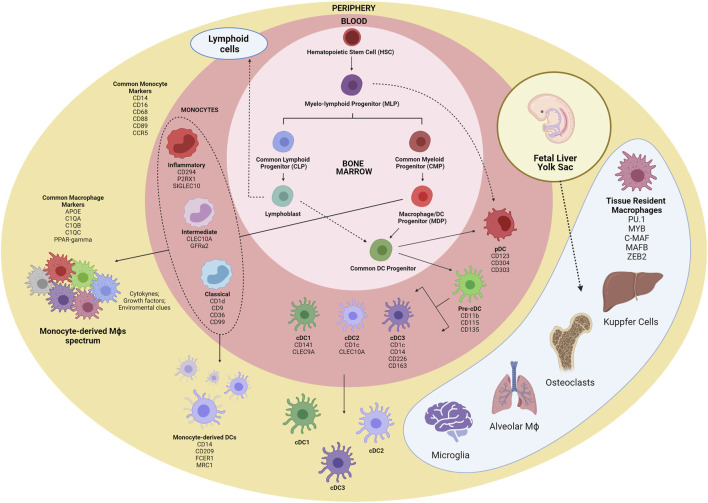
Schematic overview of myeloid cells ontogeny. The first myeloid cells arise in the embryonic phase from yolk sac-derived primitive HSCs and expand in the fetal liver, originating GMPs and blood monocytes. Tissue-resident macrophages are established before birth and are mostly repopulated by self-renewal. Within the bone marrow, CMPs give rise to GMPs and MDPs. MDPs are responsible for the contribution of many macrophages and DC subsets. MDPs differentiate into monocytes and CDPs, which in turn differentiate into pDC and pre-cDCs. Following exit from the BM, pre-cDCs enter peripheral organs and mature into cDC1, cDC2, and cDC3. Alternatively, pDCs and cDCs may also originate from MLPs. Inflammatory cues contribute to monocyte migration to tissues, where they differentiate into macrophages and Mo-DCs. HSC, hematopoeticstem-cell; GMP, granulocyte-monocyte progenitor; CMP, common myeloid progenitor; MDP, macrophage/DC progenitor; MLP, myelo-lymphoid progenitor; CDP, common DC precursor; pre-cDC, precursor of classical DC; Mo-DC, monocyte-derived DC; cDC, classical DC; pDC, plasmacytoid DC.

The first myeloid cells originate from hematopoietic progenitors in the human yolk sac at 2 to 3 weeks post conception (Ginhoux and Jung 2014; Epelman et al., 2014). Primitive hematopoietic stem cells (HSCs) enter the circulation and seed the fetal liver, giving rise to the first population of granulocyte-monocyte progenitors (GMPs) and blood monocytes (Hoeffel and Ginhoux 2015; Hoeffel et al., 2015). Under inflammatory conditions, monocytes exit to the blood and enter tissues, giving rise to subsets of macrophages and to inflammatory DCs (Auffray et al., 2009). It is noteworthy that monocytes do not give rise to cDCs and pDCs but are the main contributors of monocyte-derived DCs (Mo-DCs) during inflammation (Coillard and Segura 2019). Alternatively, commitment to the human cDC lineage can occur in early lympho-myeloid progenitors, from multipotent lymphoid progenitors (MLPs) that can give rise to monocytes, pDCs and cDCs (Helft et al., 2017; Doulatov et al., 2010) (Figure 1 **)**. Most recently, distinct groups have described a third subset of DC (also called DC3) (Dutertre et al., 2019; Bourdely et al., 2020). DC3 usually present a mixed phenotypic and functional status, between that of monocytes and cDC2, and are differentiated from a distinct pre-DC3 progenitor in a GM-CSF dependent-fashion. The DC3 subset closely resembles the monocyte-derived dendritic cells, differentiated *in vitro* from blood monocytes, and normally accumulate under inflammatory conditions.

Several studies have been conducted to unravel the origin of the terminally differentiated cells of the mononuclear phagocyte system. The analysis of cell surface markers to separate different subpopulations of cells with myeloid origin has been shown to be very limited, since there is significant phenotypic overlap in the expression of cell surface markers among these cells (Guermonprez et al., 2019). Characteristic monocyte surface markers such as CD14, CD16, CD68, and CCR5 are shared with macrophages, and the expression of general macrophage specific surface markers (C1QC and VEGF) and tissue specific markers (VCAM1 and PPARγ) are necessary for proper discrimination between monocytes and macrophages (Ginhoux and Jung 2014; Hoeffel and Ginhoux 2015).

Macrophage subsets could be further segregated into tissue-resident and monocyte-derived cells. A great variety of tissue-resident macrophages are distributed in the human body presenting specialized functional features. Distinct studies have reported *PU.1*, *MYB*, *C-MAF*, *MAF-B*, and *ZEB2* ascore transcriptional factors (TFs) shared by tissue-resident macrophages regardless of the tissue imprinting cues (Blériot et al., 2020). Under inflammatory conditions, intrinsic local signals as cytokines, growth factors, metabolites and others contribute for shaping the macrophage programing and function, generating a much more complex range of phenotypes.

The current DC classification can be quite troublesome, and there is still little consensus on the identification and naming of DC populations (Villar and Segura 2020). Nevertheless, there is a general consensus of classification of DCs into three main groups, based on their cellular and molecular ontogeny (Guilliams et al., 2014): 1) pDC; 2) conventional DC1 and DC2; and 3) DC3. The expression of the CD123, CD304, and CD303 surface markers defines pDCs, whereas CD141 and Clec9A are cDC1 specific markers, and expression of CD1c and CLEC10A seems to be restricted to cDC2. DC3/Mo-DCs share the expression of CD1c with cDC2, and CD14 with monocytes and macrophages, although they present specific subset markers, such as CD226 and CD163.

Studies on mice and *in vitro* culture of human cells have provided a better understanding of the developmental programs that seem to be hard-wired in hematopoietic progenitor cells to originate different myeloid cells (Epelman et al., 2014). These studies involved the selection of specific gene expression programs, including important TFs responsible for cell fate choices (Auffray et al., 2009; Merad and Manz 2009). The myeloid transcription factor PU.1 is required for the earliest steps of myeloid lineage commitment in HSCs (Sarrazin et al., 2009). This TF plays a role in myeloid lineage diversification, particularly during fate choice of monocytes into macrophage or DC (Bakri et al., 2005). The expression level of PU.1 in certain progenitor stages dictates the fate of a specific myeloid progenitor. Intermediate expression of PU.1 in GMPs favors differentiation to macrophages instead of granulocytes (Laslo et al., 2006). The ectopic expression of TFs Maf-B, c-Maf, Egr1, IRF8, IRF4, and PU.1 in early progenitors can drive monocyte/macrophage and DC fates, and the given function of any factor depends on cooperating or antagonistic TFs that are expressed at each specific progenitor stage (Laslo et al., 2006). According to the current classification of DCs based on ontogeny, a discussion is being raised whether certain DC populations might only be considered as distinct subsets if their developmental pathway is controlled by specific TFs (Villar and Segura 2020). Plasmacytoid DCs develop from progenitors that express the E2-2, ZEB2, IRF8, and IRF4 TFs. Conventional DC1 arise from expression of IRF8 and BATF3, while cCD2 progenitors express ZEB2, IRF4 and Notch2/KLF4 TFs. Monocyte-derived DCs generation depend on the expression of the MAFB and KLF4 TFs (Collin and Bigley 2018; Guermonprez et al., 2019). The DC3 subset developmental pathway is still a matter of discussion, although recent studies have shed some light on this ongoing debate. Cultures of purified monocytes or monocyte-committed precursors with IRF8^low^ expression were able to differentiate into DC3, while IRF8^high^ progenitors gave rise to pDC and DC2 (Bourdely et al., 2020; Cytlak et al., 2020). Besides TFs, epigenetic modification and micro-RNAs have been described to be important determinants of lineage choice (O’Connell et al., 2007; O’Connell et al., 2008).

Altogether, the possibility of generating various terminally differentiated subsets of myeloid cells *in vitro* with cytokine and growth factor cocktails can be harnessed in immunotherapy approaches to develop novel cellular therapy products with optimized biological functions (Salmon et al., 2016).

## Role of Monocytes, Macrophages and Dendritic Cells in Cancer

### The Tumor Microenvironment and its Chemoattractive *millieu*

Macrophages are critical cells that participate of tissue homeostasis and regulation and may represent up to 50% of total immune cells that infiltrate solid tumors (Ramos et al., 2020). The great majority of TAMs are derived from blood monocytes due to the chemo attractive milieu from the tumor microenvironment, constituted by a large spectrum of soluble factors that includes M-CSF, CCL2, CCL3, CCL4, CCL5, CCL8, SDF1, VEGF, MIP-1, and MIF (Panni et al., 2013). The chemokine CCL2 was extensively studied and reported as one of the key factors inducing the accumulation of circulating monocytes within tumors (Kitamura et al., 2015; Cassetta and Pollard 2018). Due to the mostly suppressive activity of TAMs, a series of reports have proposed strategies to block the CCL2-CCR2 axis aiming to avoid monocyte trafficking and accumulation into the tumor tissues. These studies revealed a significant impact on tumor growth and metastasis in pre-clinical settings (Qian et al., 2011; Macanas-Pirard et al., 2017; Nywening et al., 2018), but clinical trials failed to demonstrate similar efficacy (Fetterly et al., 2013; Pienta et al., 2013). Importantly, the restoration of the CCL2-CCR2 axis after blockage promotes new waves of monocyte recruitment and accumulation, inducing an acceleration of tumor growth on mice (Bonapace et al., 2014). In addition, no effect was noted on established tissue-resident macrophages in the tumor microenvironment after CCL2-CCR2 blockage (Zhu et al., 2017). Other molecules have been identified as promoting monocyte recruitment to tumor sites. The inhibition of CCL5-CCR5 interaction has been shown to have an impact on tumor growth and metastasis (Cambien et al., 2011; Ban et al., 2017). In fact, some studies have described CCL5 as a critical chemokine present in the tumor microenvironment that is able to promote the recruitment of DC to the tumor mass (Böttcher et al., 2018; Cueto et al., 2021). Once accumulated in the tissue, intratumoral DCs will, in turn, produce important levels of CXCL9 and CXCL10, critical chemokines that attract T cells within tumors (Spranger et al., 2017). This sequence of events may generate the stimulation of anti-tumor T cell responses and tumor control.

Targeting CXCR4, a receptor for CXCL12 molecule, has also been demonstrated to have an impact on monocyte recruitment in a colorectal tumor mouse model (Jung et al., 2017). More recently, two studies highlighted new signaling pathways able to induce monocyte/macrophage attraction and accumulation on tumors. Zhang and others showed that IFN-γ affects CXCL8-CXCR2 signaling and consequently reduced TAM trafficking within the tumor microenvironment (Zhang M. et al., 2020, 8). Takahashi et al. used *in vitro* assays to reveal the role of soluble-VCAM-1 molecule produced by pancreatic cancer cells in the chemo attraction of murine macrophages (Takahashi et al., 2020). Thus, a mixture of soluble factors may drive the migration of myeloid immune cells into the tumor microenvironment. The effective manipulation of the chemo attractive signals along with proper macrophage differentiation may promote the infiltration and accumulation of subpopulations of these cells capable of controlling the tumor.

### Monocytes

Human monocytes represent around 10% of mononuclear cells in peripheral blood. For decades, many authors have used flow cytometry to further describe these cells into three major subsets with distinct proportions: classical monocytes CD14^high^CD16^neg^ (around 90% of total monocytes), intermediate monocytes CD14^high^CD16+ (around 2% of total monocytes) and inflammatory monocytes CD14^low^CD16^high^ (around 8% of total monocytes) (Passlick et al., 1989; Ziegler-Heitbrock et al., 2010; Wong et al., 2012). Microarray mRNA analysis of FACS-sorted monocytes revealed unique transcriptional gene profiles for each of these subsets (Wong et al., 2011). Among newly described markers, authors revealed, both at mRNA and at protein levels, that classical monocytes express higher levels of CD1d, CD99, CD9 and CD36; intermediate monocytes showed higher levels of CLEC10A and GFRa2; and inflammatory monocytes showed elevated expression of CD294, P2RX1, and SIGLEC10. Additional studies using pseudo-time scRNAseq analysis have confirmed that both non-classical monocytes subsets may originate from classical monocytes, in mice and humans (Mildner et al., 2017; Villani et al., 2017). Of note, the CD163 scavenger receptor, which has been extensively used to describe macrophages subsets (Ruffell and Coussens 2015; Ramos et al., 2020), is also expressed by monocytes and newly described DC subsets (Villani et al., 2017) and is significantly more expressed in both classical and intermediate monocytes, in comparison to inflammatory monocytes. More recently, single-cell approaches have re-oriented the description of monocyte subsets by revealing new markers and functional features of these cells. Two new markers, CD88 and CD89, were described as specifically expressed by human classical monocytes, discriminating these cells from other immune cell subsets (Dutertre et al., 2019; Bourdely et al., 2020). These new findings are of great interest, considering that the characterization of monocytes in tissues is still challenging, since many of their surface markers are shared with macrophages (e.g., CD14, CD68, and CD163).

Hematopoietic stem cells give rise to progenitors that will progressively generate monocyte-committed progenitors and, subsequently, monocytes. Importantly, elevated counts of blood monocytes were described in cancer patients and tumor-bearing mice (Cortez-Retamozo et al., 2012; Sanford et al., 2013; Trovato et al., 2019), where increased numbers of blood monocytes were associated to worse prognoses (Lee, 2012; Shigeta et al., 2016; Hayashi et al., 2017; Feng et al., 2018). This phenomenon is further supported by the elevated serum levels of CCL2, a critical cytokine for monocyte mobilization from bone marrow (Monti et al., 2003; Dehqanzada et al., 2007; Sanford et al., 2013). Coherently, additional factors were found increased in cancer patients’ serum, including the classical growth factors: G-CSF, GM-CSF, and M-CSF (Scholl et al., 1996; Wu et al., 2014; Ribechini and Hutchinson 2017). Notably, M-CSF is critical during monocyte development, promoting survival and proliferation of myeloid progenitors towards monocytic cell lineages (Rieger et al., 2009). Moreover, blood monocytes under high concentrations of M-CSF may acquire an anti-inflammatory profile, giving rise to potential suppressive mature macrophages (Menetrier-Caux et al., 1998; Jaguin et al., 2013; Ramos et al., 2020).

There are emerging data indicating that tumor-derived factors can affect monocyte differentiation remotely, altering bone marrow progenitors. A series of studies have uncovered an altered transcriptomic profile of circulating monocytes in both cancer patients (Chittezhath et al., 2014; Cassetta et al., 2019; Kiss et al., 2020; Ramos et al., 2020) and mouse tumor models (Torroella-Kouri et al., 2013; Stone et al., 2014) when compared to tumor-free individuals. One important consequence of this phenomenon is the biased differentiation programming found in patients’ monocytes when compared to healthy donors, which gives rise to dysfunctional DC and macrophages (Ramos et al., 2012; Ramos et al., 2020), thus impacting adaptive anti-tumoral responses.

Due to their elevated degree of plasticity and sensitivity, monocytes can rapidly migrate and respond to inflammation in tissues. However, tumor-derived factors can also influence their process of differentiation by modulating their phenotype, differentiation and functions at tumor sites and systemically. Harnessing the great plasticity of monocytes and the precise definition of their differentiation pathways, it would be possible to recruit specifically functional cells, representing a great gain for the development of new therapies for cancer.

### Macrophages

The function and phenotype of macrophages in the tumor microenvironment have been extensively described in the last decades (Ruffell and Coussens 2015). Most of the reports have associated the features of macrophages to an oversimplified bipolar model M1 (pro-inflammatory) x M2 (anti-inflammatory) (Lacey et al., 2012; Jaguin et al., 2013). Pro-inflammatory M1-macrophages have been mostly associated to anti-tumoral responses, while M2-macrophages have shown pro-angiogenic and immunosuppressive capabilities (Pollard 2004). Based on this model, many studies have used M1 or M2-like markers to describe TAMs in distinct human tumor types. Most of the markers used included CD68, CD14, CD163, and CD206 (Steidl et al., 2010; Ruffell and Coussens 2015). CD68 and CD14 molecules are expressed by monocytes, macrophages and some DC subsets, regardless of their functional status. CD163 and CD206 markers currently correspond to an M2 immunosuppressive-like macrophages status, and are associated to poor patient prognosis for several solid and hematologic tumors, such as: breast (Medrek et al., 2012; Ramos et al., 2020), ovarian (Reinartz et al., 2014), pancreatic (Ino et al., 2013) and acute myeloid leukemia (Yang et al., 2018; Guo et al., 2021). In the recent years, with new technologies using large-scale single-cell approaches, the bipolar model of macrophage differentiation–M1 versus M2-macrophages–has been gradually replaced by a multidimensional landscape (Chevrier et al., 2017; Lavin et al., 2017; Azizi et al., 2018). Three new markers have emerged from recent studies using single-cell approaches redefining macrophage features on tumors: APOE (Apolipoprotein E), TREM2 (Triggering receptor expressed on myeloid cells 2) and FOLR2 (Folate receptor 2). APOE has been associated to a new pan-macrophage marker, distinguishing these cells from blood monocytes (Lavin et al., 2017; Azizi et al., 2018). TREM2 was described as expressed on suppressive monocyte-derived macrophages that are accumulated on tumor sites (Katzenelenbogen et al., 2020; Molgora et al., 2020; Ardighieri et al., 2021). FOLR2 molecule is expressed on tissue-resident macrophages in a variety of tissues and was also found on stromal areas from human tumors (Sharma et al., 2020; Ramos et al., 2021).

Considering that macrophages are one of the most frequent immune infiltrating cells in solid tumors, a crescent number of reports have described anti-tumor strategies that target the immunosuppressive functions of TAMs (Liu and Wang 2020). These new “omics-studies” have uncovered distinct subsets of TAM and tissue-resident macrophages presenting a complex functional programming that offer new targets for molecular targeting and new perspectives for clinical interventions.

### Dendritic Cells

Dendritic cells constitute a very heterogenous group of innate cells with specialized functions that are distributed in a wide variety of tissues. DC subsets are conserved across diverse human tumors and tissues (Gerhard et al., 2021). In contrast to other APCs, such as macrophages, B cells and monocytes, DC have a unique ability to migrate, transport and present tumor-antigens to naïve T cell in the lymphoid organs (Mildner and Jung 2014; Ruhland et al., 2020), being critical for the initiation of adaptive immune responses. A great range of tumors are devoid of DC infiltration (Broz et al., 2014), and it may explain the failure of anti-tumor T cell immunity in tumor control (Spranger et al., 2017).

Studies focused on DC biology in cancer have associated the presence of these cells with good patient outcome for distinct tumor types (Malietzis et al., 2015; Truxova et al., 2018; Melaiu et al., 2020). Not only the presence of DCs but their activation status and tissue spatial localization have highlighted the functional impact of these cells in patients. Ruffel and collaborators (Ruffell et al., 2012) have shown a decrease in frequency of tumor-infiltrating DCs in tumor areas when compared to normal adjacent tissues. Besides that, tumor-infiltrating DCs were described as dysfunctional and immature (Bell et al., 1999; Gervais et al., 2005; Dieu-Nosjean et al., 2008), and may participate in the tumor angiogenesis (Fainaru et al., 2008). In fact, DCs were also described as immature in tumor bed, in contrast to activated DCs found in tumor periphery (Treilleux et al., 2004; Baleeiro et al., 2008). A study by Goc and collaborators (Goc et al., 2014) correlated a lower risk of death in lung cancer patients with the presence of mature DCs and Th1-like lymphocytes in peritumoral tertiary lymphoid structures (TLS). Many studies have explored the tumor-induced mechanisms responsible for the blockage of DC maturation and antigen presentation for T cells. Thomachot and others (Thomachot et al., 2004) showed that products derived from human breast carcinoma cell lines were able to block the *in vitro* maturation of DC derived from CD34+ progenitors. The accumulation of some metabolites such as fatty acid and lactic acid also impair the antigen processing and presentation as well as the production of pro-inflammatory cytokines by DC (Herber et al., 2010; Cao et al., 2014; Caronni et al., 2018). In addition, a variety of cytokines were reported as critical for dampening DC functionality. The vascular endothelial growth factor (VEGF) is highly expressed in the tumor microenvironment from distinct tumor types and inhibit the activity of FMS-like tyrosine kinase 3 ligand (FLT3L), a critical factor for DC development and maturation (Gabrilovich et al., 1996). IL-6 and IL-10 are known to play a critical role in the blockage of DC functions by promoting STAT3 activation, which may impede IL-12 production and promote IL-10 amplification (Diao et al., 2011; Tang et al., 2015). Farren and others (Farren et al., 2014) suggested that tumor-derived factors sustained STAT3 up-regulation on myeloid cells progenitors avoiding the activation of ERK and NF-kB signaling, which may limit the monocyte capacity to be differentiated into DCs. Furthermore, TGF-β present in the tumor microenvironment has been reported as critical to suppress DC maturation and inhibit their production of pro-inflammatory cytokines such as IL-12 and TNF-α, hampering the activation of T cells (Belladonna et al., 2008; Flavell et al., 2010). Importantly, tumor factors may also affect myeloid APCs at circulation, since defective subpopulations of DCs were described in the blood of cancer patients (Satthaporn et al., 2004).

Recent reports using transgenic mice models and single-cell approaches have further detailed the intrinsic features of the distinct DC subsets in the oncology field. Both mouse and human cDC1 were described as highly efficient for cross-presentation in the tumor context by stimulating CD8+ T cells responses (Jongbloed et al., 2010; Wculek et al., 2019). Additional studies have described that type I interferon may enhance anti-tumor properties of cDC1 cross-presentation and CD8+ T cell stimulation (Diamond et al., 2011; Fuertes et al., 2011). The cDC2 subset constitute the most heterogenous population of conventional DCs presenting a wide diversity of phenotypes (Villani et al., 2017; Dutertre et al., 2019). These cells can produce a large spectrum of cytokines and are involved in the induction of diverse CD4+ T helper subsets in both inflammatory and cancer contexts (Binnewies et al., 2019; Bosteels et al., 2020). The recently described DC3 is still under deep characterization. A study by Bourdely and others (Bourdely et al., 2020) have described the DC3 subset as effective for the priming of CD8+CD103+ tissue-resident memory T cells via the production of TGF-beta. In agreement, presence of DC3 are also associated to the accumulation of CD8+ T tissue-resident memory cells in human breast cancer tissues. Dutertre and collaborators (Dutertre et al., 2019) also reported that DC3 are capable to expand CD4+ T helper cells *in vitro*. Of note, CD14+CD163+ DC3 subset were strong inducers of CD4+IL17A+ Th17-likecells when compared to cDC2 subtype, suggesting their role in inflammatory responses.

The plasmacytoid DC subset has been extensively characterized in the tumor field. These cells were associated to a poor patients’ prognosis in distinct types of cancer (Wculek et al., 2020). In fact, various reports have described a tolerogenic profile of tumor-infiltrating pDCs. Breast cancer infiltrating pDCs have shown an impaired capability to secrete IFN-α (Sisirak et al., 2012), which may be partially explained by the higher concentrations of TGF-β and TNF-α found in the tumor microenvironment (Sisirak et al., 2013). Similarly, the dysfunctional pDCs profiling was described for other tumor types including ovarian (Labidi-Galy et al., 2011) and melanoma (Camisaschi et al., 2014). Other studies also uncovered the pDC role in the induction of suppressive CD4+ regulatory T cells in breast cancer patients, suggesting the mechanisms that explain their pro-tumorigenic profile (Faget et al., 2012; Sisirak et al., 2012).

Another critical DC subset described in many inflammatory conditions, including cancer, is the monocyte-derived DC. These cells can be found in the tissues from both mice and humans and emerge via the recruitment of CCR2+ monocytes from the blood (Schlitzer et al., 2015). *In vivo*, is still challenging to exactly define Mo-DCs phenotype and function due to their high plasticity and its profiling that overlaps with other DC subsets and macrophages (Collin and Bigley 2018). Many reports have described strategies for the manipulation of Mo-DCs due to the possibility of Mo-DC *in vitro* differentiation from blood monocytes (Sallusto and Lanzavecchia 1994) or CD34+ progenitors (Caux et al., 1996). It has allowed researchers to develop clinical protocols using Mo-DC based-vaccines to treat distinct diseases, including cancer (Barbuto et al., 2004; Neves et al., 2005). Mo-DCs plasticity can be also explored by treating monocytes with soluble factors, cytokines, TLR-L and other compounds to drive their cellular functions towards the stimulation of a variety of T helper responses and/or CD8+ T cell activation (Harris 2011; Goudot et al., 2017). *In vivo*, Mo-DC has been associated to an anti-tumor function. Kuhn and collaborators (Kuhn et al., 2015) reported that the blockage of Mo-DC recruitment and differentiation in tumor sites impede the stimulation of anti-tumor immunity in mice models. In addition, Mo-DCs isolated from cancer patients are capable to cross-present tumor antigens and activate cytotoxic CD8+ T cells *ex vivo* (Tang-Huau et al., 2018)*.*

Altogether, DCs critically participate in the initiation and expansion of anti-tumor adaptive immune responses with specialized functions and intrinsic roles for each subset. Despite all the uncertainty, however, DC continues as a central piece in tumor immunotherapy. From one side, the effectiveness of other immunotherapeutic modalities highlights the immune system’s effector mechanisms potential in cancer therapy and DCs remain the best-known cells to initiate the immune responses that would recruit such mechanisms.

Another fact that should be considered when looking at DC in the context of immunotherapy is, from one side, the heterogeneity and plasticity of the immune response and, from the other, our still incomplete knowledge of its role in each disease. Although improving the scenario is still uncertain. However, the use of a cell, itself heterogeneous and plastic, and that is hardly constrained by our manipulations, leaves the door open to “unexpected” results, which might become those that actually point to the “right” way to achieve success. The comprehensive use of these cells by exploring their skills and improving their clinical application alone or in combination with other therapies may represent a great gain for cancer patients’ treatment.

## Chimeric Antigen Receptor on T Cells and Natural Killer Cells

The development of T cells expressing CAR date from the first studies performed in the 80’s by Gross and collaborators (Gross et al., 1989). This strategy was further optimized in the subsequent years by the comprehension of the critical mechanisms for CAR-T cell activation, expansion and *in vivo* survival (Krause et al., 1998; Maher et al., 2002; Brentjens et al., 2003). The first trials using genetically engineered T cells highlighted its safety and its impact on cancer patients’ survival (Morgan et al., 2006; Kochenderfer et al., 2010), paving the way for the subsequent clinical applications. Since 2017, the Food and Drug Administration department (FDA, United States) has approved five products of CAR-T for refractory non-Hodgkin B cell lymphoma, acute lymphocytic leukemia and multiple myeloma (Maude et al., 2018; Park et al., 2018; Munshi et al., 2021). Lately, clinical trials using CAR-T cells have been really encouraging with studies reporting residual disease-negative complete responses for about 70% of patients with distinct hematological malignancies (Ortíz-Maldonado et al., 2021). More recently, studies have also focused on the improvement of the manufacturing process of CAR-T cells by aiming to diminish the time for production and costs, while ameliorating the quality and the life-spam of the cells *in vivo*. The creation of the 4th generation (Chmielewski and Abken 2020) and bispecific CAR-T cells (Shah et al., 2020; Fousek et al., 2021) are examples of such improvements to be applied in the clinics.

Another recent strategy was the manufacture of NK cells expressing CAR. Many advantages were considered in the use of these cells to treat malignances, including: its intrinsic ability to eliminate cells with down regulated HLA expression, its application in allogenic scenarios and a lower potential of toxicity, which will reduce side-effects and costs with patients’ conditioning (Moretta et al., 2011; Simonetta et al., 2017; Xie et al., 2020; Albinger et al., 2021). Pre-clinical reports have demonstrated that CAR-NKs generated from both peripheral blood and umbilical cord blood have effective capabilities to eliminate CD19+ target tumor cells *in vitro* (Herrera et al., 2019). In the clinical settings, trials using cord blood-derived CAR-NK for patients with B-cell malignancies showed very enthusiastic results, by reporting clinical responses in around 70% of treated individuals and CAR-NK persistence at the periphery for around 1 year (Liu et al., 2020).

Despite great success of CAR-T and CAR-NK treatment for hematological cancers, first clinical trials using CAR-T cells for solid tumors have reported disappointing results (Kershaw et al., 2006; Lamers et al., 2011; Thistlethwaite et al., 2017). Most clinical trials using CAR-NK for solid tumors are still ongoing and no conclusive results were presented so far. Many authors have extensively discussed the possible limitations for the success of CAR-T and CAR-NK in solid tumors (Hanahan and Coussens 2012; Li et al., 2018; Marofi et al., 2021). Main cited reasons include but are not limited to: the high mutational burden generating a diversity of tumor antigens, the dense extracellular matrix that may promote T cell exclusion, the absence of chemo-attractive factors for T cells, the limited *in vivo* persistence of CAR-T cells and the immunosuppressive microenvironment.

## Chimeric Antigen Receptor on Myeloid Immune Cells

**FIGURE 2:**
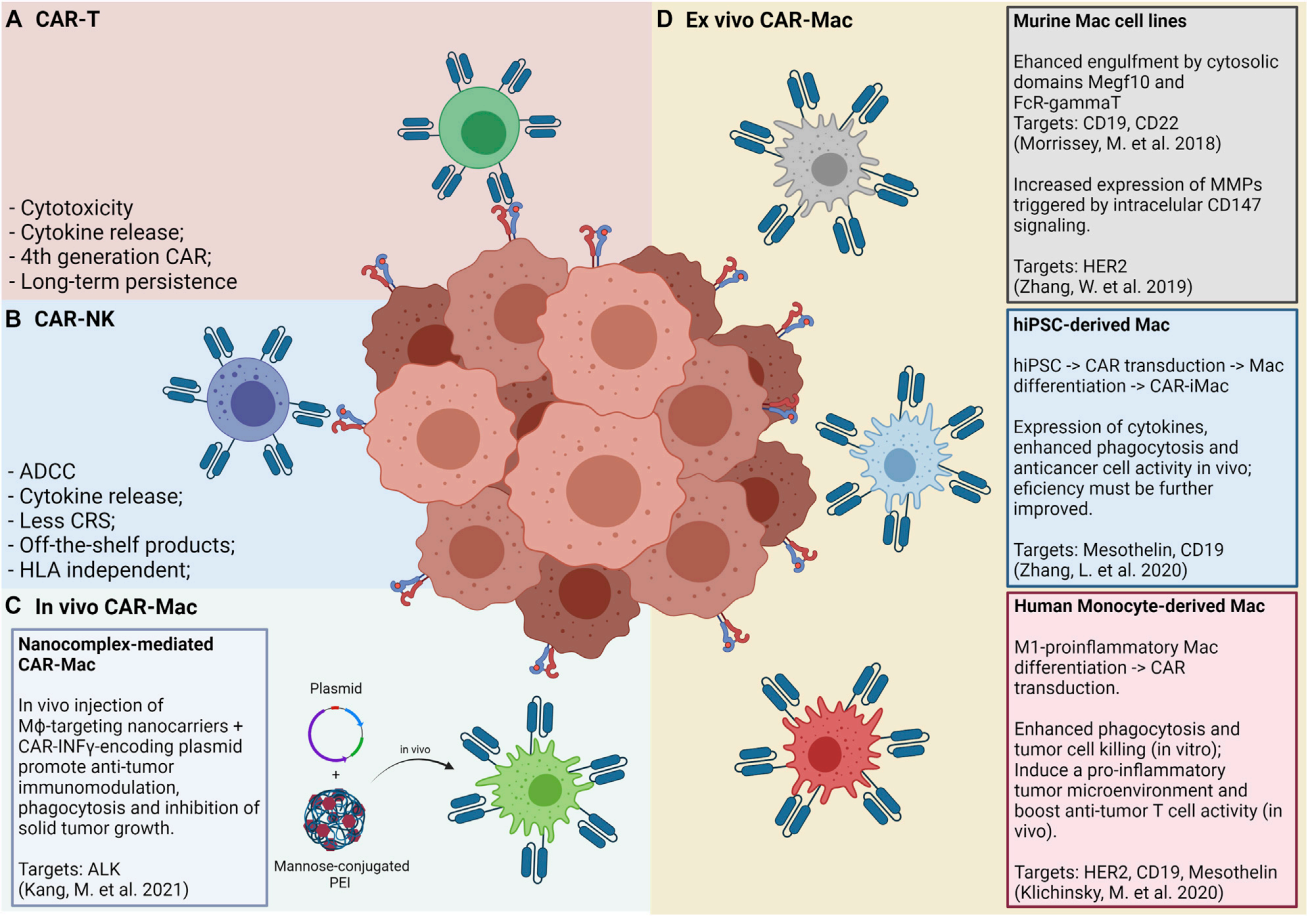
Panels **(A)** and **(B)** show the “classic” CAR-T and CAR-NK approaches, respectively, and its main features and advantages. Panel **(C)** shows an *in vivo* approach for CAR-Mac production, in which a nanocomplex composed of mannose-conjugated PEI and DNA plasmids is intratumorally or intraperitonially administered and internalized by local macrophages. Panel **(D)** shows the approaches so far tested for generating and applying CAR-Mac cells against solid tumors (Morrissey et al., 2018; Zhang et al., 2019; Klichinsky et al., 2020; Zhang L. et al., 2020). Both murine and human macrophages were reprogrammed to target at least four tumor markers (CD19, CD22, HER2, and Mesothelin). Human macrophages were obtained from peripheral blood monocytes and from iPS cells.

The low efficacy of CAR-T cell therapies for solid tumors may be, probably, explained by the inability of these cells to effectively infiltrate the tumor mass. Among the main reasons for this failure are the lack of classical T-attractive chemokines (e.g., CXCL9 and CXCL10), the dense tumor microenvironment matrix and the higher tumor-antigen heterogeneity, as compared to hematologic tumors. To overcome these difficulties, recent studies have suggested the use of CAR-engineered monocytes/macrophages for the elimination of solid tumors. Still, few reports have described CAR-Mac functionalities, but this emerging topic has called the attention of the scientific community. We summarize below the key works recently published using CAR-Mac technologies highlighting their particularities (Figure 2).

The study by Morrissey and collaborators (2018) (Morrissey et al., 2018) used murine CAR-Mac constructs strategies to enhance the phagocytosis of cancer cells. The authors’ strategy consists in an extracellular single-chain antibody variable fragment (ScFv) specific for CD19 or CD22 with the CD8 transmembrane domain linked to a cytoplasmatic domain able to promote macrophage phagocytosis. By screening a library of intracellular domains of engulfment receptors, the authors selected the cytosolic domains from Megf10 and FcR-gamma, achieving a robust engulfment of antigen-coated synthetic particles. Macrophages expressing anti-CD19 or anti-CD22 CARs linked with the cytosolic domain Megf10 showed superior abilities of phagocytosis of synthetic beads with up to 20 µm of size when compared to CAR-GFP controls. Authors further demonstrated that the synapse of CAR-Mac cell-bead interaction induced stimulation via phosphotyrosine expression at comparative levels of CD3ζ signaling. Authors next tested the phagocytic capabilities of J774A.1 CAR-Mac expressing Megf10 and FcRγ intracellular domains in co-culture with human Raji B cell lines, cells that express CD19 endogenously. Elegant images showed that CAR-Mac with both intracellular domains presented a significant higher capability to promote trogocytosis or to engulf tumor cells when compared to control CAR-Mac GFP+. This effect was further improved when the authors engineered CAR-Mac to increase the PI3K employment by fusing the CD19 cytoplasmatic domain, which is able to recruit the p85 subunit of PI3K. Altogether, this study provides an attractive engineered strategy to increase engulfment and elimination of tumor cells by CAR-Mac.

Another interesting study reported by Zhang and others (2019) (Zhang et al., 2019) described a murine anti-HER2 CAR-Mac Raw264.7 engineered with an intracellular signaling domain of CD147 (CAR-147-Mac), able to trigger the expression of matrix metalloproteinase (MMPs) after antigen recognition. This strategy aims to remodel the tumor microenvironment extracellular matrix, facilitating the infiltration of immune cells or drugs. Authors showed that CAR-147-Mac were able to produce significant high levels of mRNA for distinct MMPs upon HER2 antigen binding in co-cultures with 4T1 cell lines during 24 and 48 h. A more pronounced expression of *MMP3, MMP9, MMP10*, and *MMP13* was noted when compared to co-cultures between 4T1 cell lines lacking HER2 antigen expression. For *in vivo* assays, CAR-147-Mac were found in liver and tumors of mice even after 7 days of intravenous infusion. No differences were noted in total body weight of HER2+ 4T1 tumor-bearing mice injected with both CAR-147-Mac or control CAR-Mac, but significant reduction of the weight of the spleen and tumor mass was noted in animals that received CAR-147-Mac. The infusion of CAR-147-Mac significantly inhibited HER2+ 4T1 tumor growth in mice along with an up-regulation of the anti-tumor cytokines such as IL-12 and IFN-gamma, thus suggesting that this strategy involves both local modulation and systemic anti-tumor responses. In addition, CAR-147-Mac treatment promoted an increase in T-cell infiltration and a reduction of GR1+ myeloid cells into tumors. Added, no differences were found in the infiltration of DCs, NKs, and TAMs as well as in markers of activation for T cells (PD-1, CD44, CD62L, and CD107a) when comparing tumor-bearing mice that received CAR-147-Mac or control CAR-Mac. In agreement, authors used balb/c nude mice model to demonstrate that no effect in tumor control was noted after CAR-147-Mac infusion. By showing IHC images from tumor sections authors reported a significant reduction in tumor collagen deposition in mice treated by CAR-147-Mac. Using a human *in vitro* 3D spheroid system by co-culturing THP CAR-147-Mac with HER2+ MDA-MB-453 tumor cell line authors showed an increased mRNA expression of various MMPs allowing the infiltration and accumulation of T cells. In conclusion, this study provided an interesting approach using engineered macrophages able to modify the extracellular matrix, allowing T cell infiltration in the tumor mass.

More recently, two additional studies reported strategies for CAR-Mac manufacturing using human cells as primary resources. Zhang and collaborators (Zhang L. et al., 2020) focused on undifferentiated stem cells by developing a three-step procedure: I) reprogramming human PBMCs towards an induced pluripotent stem cell (iPSCs) using non-integrating episomal vectors; II) engineering CAR expression on IPSCs by lentiviral transduction; III) promoting macrophage differentiation from CAR-iPSCs using a cocktail of growth factors and cytokines including bFGF, VEGF, SCF, IGF-1, IL-3, M-CSF, and GM-CSF to obtain a final cell product of CAR induced macrophages (CAR-iMac). Authors firstly characterized and validated CAR-iMac phenotype at both transcriptional and protein levels. By flow cytometry iCAR-Mac closely resembled to primarily differentiated macrophages by showing the expression of typical proteins, such as CD11b, CD14, CD163, and CD68. At the transcriptional level CAR-iMac displayed a distinct programing from those undifferentiated IPS and clustered with primary differentiated macrophages. In addition, CAR-iMac present enrichment for genes related to GO pathways ofinnate immunity, antigen processing and presentation and positive regulation of cytokine production while expressing high levels of characteristic macrophage genes such as*AIF1*, *CSFR1*, and *SPI1.* Authors also implied scRNA-seq analysis and found that CAR-iMac cells clustered with macrophages already described in public datasets. Subsequently, authors aimed to validate functional features of iCAR-Mac against two antigens, CD19 and mesothelin. Anti-CD19 CAR-iMac co-cultured with CD19+ K562 cells expressed higher levels of *TNF*, *IL1A*, *IL1B*, and *IL-6* and displayed enrichment on pathways related to antigen process and presentation and positive regulation of cytokine production when compared to control CD19- K562 cells. Authors also showed an increased phagocytosis of anti-CD19 CAR-iMac when K562 target cells expressed CD19. Similar results were obtained for *in vitro* assays using Meso+ OVCAR3 cells lines. For *in vivo* assays, NSG mice were injected with luciferase-expressing Meso+ HO8910 cell lines intraperitoneally, prior to anti-meso CAR-iMac infusion. Data showed a significant reduction in bioluminescent tumor cells when compared to PBS-treated mice at days 4, 11, and 14 post-treatment. Considering that pluripotent stem cells have a great expansion potential it could be maintained as an unlimited source. Thus, the platform for iPSC-derived engineered CAR-Mac emerges as a very promising approach to be further explored for a variety of malignancies and tumor antigens.

An elegant study by Klichinsky and others (Klichinsky et al., 2020) reported the engineering of CAR-Mac from human monocyte-derived macrophages applying the classical M1-like *in vitro* protocol based on GM-CSF treatment. Moreover, the authors established a new protocol for primary macrophage transduction with high reproducibility (validated in more than 10 distinct monocyte donors) and efficiency (>75% of CAR-positive cells) by using the chimeric adenoviral vector Ad5f35. CAR-Mac manufactured with Ad5f35 adenovirus were able to efficiently phagocyte tumor cells from both hematological and solid tumor lines as well as control tumor growth in xenograft mouse models when administered via intravenous or intraperitoneal routes. Interestingly, CAR-Mac transfected with Ad5f35 showed superior phagocytic capacity than control M1-like macrophages and presented a stable pro-inflammatory phenotype in the tumor microenvironment. CAR-Mac were also found infiltrating distinct organs from tumor bearing-mice and persisted for up to 62 days in tumor-free animals. Surprisingly, the authors also reported efficient cross-presentation promoted by CAR-Mac transfected with Ad5f35, since in co-cultures with CAR-Mac pre-incubated with NYESO1+ tumor cell lines in an HLA-A201+ restricted context, an increase in NYESO1+CD8+CD69+ activated T lymphocytes and an up-regulation of IFN-gamma secretion was detected. In addition, CAR-Mac were able to efficiently recruit both resting and activated T cells in chemotaxis assays and to induce activation of immature DCs via soluble factors. Importantly, this study by Klichinsky and others (Klichinsky et al., 2020) paved the way for the first phase-I clinical trial using CAR-Mac launched in 2020 for a variety of HER2-overexpressing solid tumors (NCT04660929). In this study, the authors estimate the recruitment of patients with no available curative treatment options that will be separated into two treatment groups. One group will undergo intra subject dose escalation of IV administrations of up to 500 million cells on D+0, up to 1.5 billion cells on D+3, and up to 3 billion cells on D+5. The other group will receive a single dose of up to 5 billion cells on D+0. As primary endpoints, the investigators will evaluate the safety and tolerability of the infused product by estimating the frequency and severity of adverse events (i.e., CRS) in the trial subjects during a 14 months follow-up period. The feasibility of manufacturing anti-HER2 CAR-Mac will also be assessed by describing the percentage of products that pass the release criteria. The Overall Response Rate (ORR), as well as the Progression Free Survival (PFS) of subjects that receive at least one dose of the product will be assessed as secondary endpoints within a time frame of 24 months.

More recently, Kang and collaborators (Kang et al., 2021) have described an *in vivo* approach for CAR-Mac reprograming using mannose-conjugated polyethylenimine (MPEI) nanocomplex as a gene delivery of plasmids for anti-ALK (anaplastic lymphoma kinase) CAR and IFN-gamma. Based on the fact that TAMs with anti-inflammatory properties may overexpress the mannose receptor (CD206), authors took advantage of this system to target macrophages *in situ.* They added in the plasmid construction the *IFN-γ* gene, a classical known cytokine able to stimulate a pro-inflammatory phenotype (Ramos et al., 2020). Both ALK-CAR and IFN-γ genes were cloned using the non-viral transposon system of piggyBac, for stable and persistent CAR expression. Using this systems authors proposed to target ALK tumor-antigen as well as to reprogram TAMs to acquire pro-inflammatory properties inducing adaptive immune T cell responses. Authors firstly validated the manufacturing of MPEI/pCAR-IFN-γ nanocomplexes indicating homogenous sizes and a great stability in serum for up to 7 days. Also, no cytotoxicity was noted in treated-bone marrow derived macrophages (BMDM), whereas CAR and IFN-gamma expression were validated in *in vitro* treated M2-BMDM. Of note, the efficiency of transfection was about 14% in M2-BMDM treated cells with a 20-fold increasing in IFN-gamma expression. In terms of functional *in vitro* assays, authors reported an increase of phagocytosis capability of M2-BMDM MPEI/pCAR-IFN-γ treated cells for distinct ratios of BMDM: ALK+ target Neuro-2a tumor cell. In addition, M2-BMDM presented a shift of phenotype towards a M1-like profile once treated by MPEI/IFN-γ or MPEI/pCAR-IFN-γ by showing a significantly down-regulation of CD163, CD206, IL-10, and arginase-1 concomitantly with an up-regulation of CD80, CD86, and TNF-alpha. Subsequently, authors tested the *in vivo* effects of MPEI/pCAR-IFN-γ and variations of this construction in tumor growth and tumor microenvironment modulation. Using a Neuro-2a syngeneic tumor-bearing mice model, authors reported a significant control of tumor growth in animals receiving both MPEI/pCAR or MPEI/IFN-γ, but this anti-tumoral effect was further increased under intratumoral infusion of the MPEI/pCAR-IFN-γ full construct. By analyzing the tumor microenvironment composition after MPEI/pCAR-IFN-γ treatment, authors found a significant increase in CD8+ T cell infiltration and INOS expression along with a strongly reduced expression of Arginase-1, TGF-beta, and IL10, indicating local immune modulation effects. Alternatively, by injecting MPEI/pCAR-IFN-γ intraperitoneally in tumor-bearing mice authors showed presence of CAR+ immune cells in liver, kidney and tumor mass as well as a significant effect on tumor control, even considering it less pronounced than intratumoral infusion. In conclusion, the transfection efficiency observed in this study was lower than that of lentiviral transduction and should be further improved. Importantly, *in vivo* infusion of MPEI/pCAR-IFN-γ nanocomplex allows the transfection of a variety of immune cells, including DCs and T lymphocytes, possibly amplifying the anti-tumoral effects. Thus, this nanocomplex strategy may overcome the high costs, the exhaustive process for CAR-Mac manufacturing and avoid the concerns of the use of viral vectors representing an important new avenue for future strategies.

## Limitations of CAR-Myeloid Cells

We have summarized in Table 1 the main differences among CAR-T, CAR-NK, and CAR-myeloid cells by highlighting the process for manufacturing of these cells and their potential applications. Two important obstacles of using myeloid cells in CAR-based therapies are the inability of primary macrophages and DCs to expand *in vitro* and the difficulty in delivering exogenous genetic material (Keller et al., 2018). Viral and non-viral delivery systems were tested in macrophages and DCs in the past decades and both presented limitations. Professional phagocytes present a powerful immune response against viral DNA making it difficult to efficiently deliver a transgene through viral vectors. On the other hand, the application of physical transfection such as nucleoporation or nucleofection did not elicit important immune responses but caused important changes in gene expression profile and functional status of macrophages and DCs (Harizaj et al., 2021). As a matter of comparison, the main steps of CAR-T cell production involve the reprogramming and expansion of T cells in order to obtain a high number of CAR+ cells before infusion. New studies have described the use of artificial APCs to stimulate T cells. The distinct approaches include cell lines modified to provide antigen-presentation and co-stimulation (Couture et al., 2019; Schmidts et al., 2020) and synthetic complexes of mesoporous silica microrods loaded with soluble mitogenic compounds and T-cell ligands (Zhang D. K. Y. et al., 2020). Recent studies have also described strategies to stimulate NK cells based on cytokine cocktails, generating a memory-like phenotype (Pahl et al., 2018). These cells present a long-lived profiling by expanding and producing IFN-gamma some weeks post-infusion. Considering that macrophages and DCs do not have the same potential of expansion as compared to T or NK cells, the number of CAR-Mac or CAR-DCs available for infusion would be significantly lower than in CAR-T or CAR-NK applications, which may limit their use in large tumors or in strategies using multiple doses.

**TABLE 1:** Comparison of key manufacturing steps for CAR-based cellular products (CAR-T, CAR-NK, and CAR-Myeloid cells) and its specific advantages and disadvantages. PEI, Polyethylenimine; GM-CSF, Granulocyte-macrophage colony-stimulating factor; M-CSF, Macrophage colony-stimulating factor.

	CAR-T cells	CAR-NK cells	CAR-myeloid cells (CAR-Mac, CAR-DC)
1. Cell type and selection	Positive selection of CD3+ and/or CD4+ or CD8+ cells	Positive selection of CD56+ cells	Positive or negative selection of CD14+ cells
2. Activation/differentiation	Beads/antibodies anti-CD3/CD28	Beads/antibodies anti-CD335/CD2	Culture with GM-CSF, M-CSF, TNF-alpha, IFN-gamma
	Artificial APC cell lines	Culture with IL-2, IL-15 and/or IL-21	TLR-Ligands
3. Gene delivery			
3.1 CAR viral delivery	Retroviral vector	Retroviral vector	Adenoviral vector
	Lentiviral vector	Lentiviral vector	Lentiviral vector
3.2 Non-Viral CAR delivery	Nucleoporation	Nucleoporation	Nanocomplex (Mannose-associated PEI + DNA plasmid)
	Liposomes	Liposomes	Expression systems
	Expression systems	Expression systems	PiggyBac
	Sleeping Beauty; PiggyBac; mRNA	Sleeping Beauty; PiggyBac; mRNA	
	Nanoplasmids	Nanoplasmids	
4. Expansion	Culture with IL-2, IL-7, IL-15 and/or IL-21	K562 feeder cells expressing membrane bound IL-21	Primary human macrophages cannot be expanded *ex vivo*
		Culture with IL-12 and IL-15	THP-1 cell line spontaneously expands *in vitro* and can be differentiated when cultured with PMA.
		Memory-like phenotype: IL-12, IL-15, and IL-18	
5. Final Product			
5.1 Lifespan	Long-term persistence after tumor remission	Mid-term lifespan *in vivo*	Short-term lifespan of myeloid cells *in vivo* (no available data for CAR-Myeloid)
5.2 Advantages	Efficient selection, activation and easy handling	Applicable in allogenic scenarios	Applicable in allogenic scenarios
		Low toxicity	Low toxicity in animal models
		Can be obtained from multiple sources	Potential application in solid tumors
			CAR-DCs may present a migratory potential to lymphoid organs
5.3 Disadvantages	Long-term cultures and/or high doses of IL-2 can induce terminally differentiated/exhausted T cells or biased expansion of regulatory CD4+ T cells	Few cells are obtained after selection	Laborious transfection procedures
	Cytokine release syndrome (CRS) and/or Immune effector cell associated neurotoxicity syndrome (ICANS) may occur	Laborious expansion and genetic modification	Difficult genetic reprogramming due to the recognition of foreign genetic material
	Long-term “on-target, off-tumor” effects (ex.: B cell aplasia when targeting CD19)		No results from clinical trials so far

Besides, the relatively short lifespan of CAR-Mac compared to CAR-T cells and CAR-NKs is a topic of concern. As discussed above, first studies are encouraging, but additional pre-clinical tests are needed to evaluate the functional status, the persistence of CAR-Mac *in vivo*, as well as the variation of cell number needed for infusion. To assure that CAR-Mac will reach the tumor mass in the shortest possible time after infusion, diverse routes of injection should be further explored in tumors of distinct origins since tissue arquiteture may vary considerably. So far, few targets have been tested in the use of pre-clinical CAR-Mac and clinical translation of CAR-myeloid cells and the future of its application relies on the overcome of technical obstacles.

## Concluding Remarks

Myeloid immune cells engineered to express CAR opened a new pathway to treat cancer that, apparently, bypasses the limitations of CAR-T or CAR-NK cells. Additional advantages of myeloid cells should be further explored and combined with CAR-engineering. In addition, the lack of response to a checkpoint inhibitor would be better explained by the absence of a response to be released from its inhibitors and, thus, DC and macrophages associated to CAR expression would be ideal candidates to add to checkpoint inhibition, a combination that is already under investigation (Wilgenhof et al., 2016). We highlighted below some key points to be considered in the use of CAR-expressing myeloid cells for solid cancers:

I) Migratory ability: monocytes and DC subtypes have great migratory skills towards a variety of inflamed and/or lymphoid tissues. The manipulation of these features needs to be further enhanced to drive these cells into specific tissues or tumor mass in a more controlled way.
II) Plasticity potential: numerous protocols of differentiation and activation may allow a better phenotypical and functional fine-tuning adjustment of monocytes, macrophages and DCs to be adapted for distinct tumor contexts and tissues. The combined use of growth factors, cytokines/chemokines, TLR-L and drugs may generate a more effective and functional manufactured CAR-myeloid cell.
III) Phagocytic skills: macrophages and DCs have unique abilities of phagocytosis and clearance for a variety of particles. The manufacturing of cells with an improved engulfment ability to eliminate tumor cells may represent a great gain in the control of malignances.
IV) Tissue remodeling: monocytes/macrophages could be reprogrammed to release a series of enzymes, cathepsins, MMPs and other modulatory factors not only to remodel the tumor microenvironment matrix but also to attract, allow the infiltration and stimulate T cells.
V) Stimulation of adaptive immunity: myeloid immune cells are capable of efficiently present tumor antigens, provide co-stimulatory signals and produce cytokines able to efficiently stimulate lymphocyte activation and expansion.

There is an emergent need to evaluate CAR-myeloid immune cells in distinct pre-clinical tumor types, considering intrinsic tissue-related factors, matrix architecture and cellular composition. Tumor growth and its intrinsic features deeply vary among models and, possibly, additional adjusts should be implemented in the manufacture of CAR-Mac. Importantly, the use of myeloid cells expressing CAR could be further combined with other clinical approaches able to disrupt the compact matrix of solid tumors and may represent a great gain for patients’ outcome. These therapies include, but are not limited to, chemotherapy, radiotherapy, oncolytic virus and/or monoclonal agonistic antibodies.
